# Following the giant’s paces-governance issues and bioethical reflections in China

**DOI:** 10.1186/1472-6939-15-79

**Published:** 2014-10-31

**Authors:** Zhaochen Wang, Di Zhang, Vincent H Ng, Reidar Lie, Xiaomei Zhai

**Affiliations:** School of Humanities and Social Sciences, Peking Union Medical College, Dong Dan San Tiao #9, Dongcheng District, Beijing, 100730 People’s Republic of China; Center for Bioethics, Chinese Academy of Medical Sciences & Peking Union Medical College, Dong Dan San Tiao #9, Dongcheng District, Beijing, 100730 People’s Republic of China; Department of Mathematics and Sciences, Northern Virginia Community College, 15200 Neabsco Mills Road, Woodbridge, VA 22191-4099 USA; Department of Philosophy, University of Bergen, Sydnesplassen 12, Bergen, Norway

**Keywords:** Biosamples, Data, BGI, Ethics, Governance, China

## Abstract

**Background:**

China has become a global player in the field of biosamples research and analysis of genetic data. The Beijing Genomics Institute is a genetics factory where enormous amounts of biosamples/data from all over the world are being analyzed. Most of the global bioethics discussions focused on research conducted by scientists from industrialized countries with subjects from poorer countries. Today, however, samples from industrialized nations are being analyzed in China on an unprecedented scale. This means that one should not just focus on bioethics developments in western countries, but also should pay attention to the situation in China. Under this era of rapid advancement in genomics, reassessing the conventionally accepted bioethical principles is strongly needed.

**Discussion:**

In this paper, we will analyze the case of BGI in the context of the Chinese regulatory system in order to identify methods to regulate genetic research more effectively and to strengthen BGI’s role in international collaborative research projects. Three main issues concerning sample collection and samples/data management are addressed. Firstly, an ambiguous definition of research, which does not specifically include biosamples/data, when applied to genetic research, may cause confusion and leave loopholes in governance. Secondly, the current regulations do not provide sufficient guidelines on the details of what information to present to prospective subjects, and how to combine informed consent with strategies of re-consent, withdrawal and feedback from research. Finally, the existing regulations do not adequately address issues of genetic privacy and data protection.

**Summary:**

Bioethical issues related to genetic research in China may be partially due to the nature of genetic research and partially stems from the strategy of simply adopting general international guidelines into the Chinese context without detailed considerations of the local needs. However, there are no perfect readymade ethical solutions for everyone; every country faces different open questions and challenges behind what appears to be unified guidelines. Given the importance of China in international genetic research, other countries ought to be concerned about the bioethical developments in China. China should also have a substantive discussion with the international community on bioethics issues.

## Background

The Beijing Genomics Institute (BGI) is an internationally renowned institute conducting research at the forefront of science with multitudes of cutting-edged equipment. It has surprised the world with its rapid growth and its wide expanding business scope. Given the success of institutions like BGI, China has now become a world player in the field of genome sequencing and a hugely influential powerhouse in international genetic research collaborations [1].

However, along with the praise, there are also questions and queries. The concerns arise not only because of BGI’s scale and capacity as a powerful global genetics factory, but also because of worries about the Chinese regulatory system lagging behind the striding pace of this genetic giant. This makes BGI an ideal case for this paper to reflect on the practical governance and bioethical issues facing genetic research in China.

### BGI’s blueprint and noted research

This paper will use BGI as a point of departure to reflect on some crucial bioethical issues in the existing system of governance of biomedical research in China. We will mainly focus on some highlighted concerns about existing regulations on genetic research, in particular on how to provide information to research subjects, and the issues of privacy and data protection.

BGI has presented its development blueprint in a published interview [2]. It consists of four parts or phases.

The first part is BGI’s own scientific research. In 1999, when geneticists from the US and other countries gathered and embarked on the Human Genome Project (HGP), the initial research group of BGI for the first time proposed that China should join in the project and later undertook 1% of the workload. After the HGP, BGI undertook 10% of the workload of the International Human HapMap Project (HapMap) in 2002 [3]. In 2008, they took part in the International 1000 Genomes Project (1000 Genomes) and the International Cancer Genome Project (ICGC Project). BGI’s research capacity and quality of research are highly noteworthy. From 2001 to 2013, around 250 papers have been published in first-class journals [4]. With more than one hundred top-notch sequencers and a strong dedicated team of researchers, BGI research groups are strongly competitive which attracts further research and development cooperation from scientific counterparts, pharmaceutical industries, and hospitals. This also gives BGI strong credence to apply for funding from the government and other sources.

The second part is the sequencing and analysis service for scientific and technological institutions and pharmaceutical companies. This part of the business now earns the company over one billion RMB in income per year, and has become the mainstay of their economic resources. It is estimated that the income could increase tenfold in the future [2].

The third part is medical/clinical services collaborations with hospitals such as analyzing clinical samples. This part may also draw huge attention from investors, and is expected to continue to generate substantial revenue and economic benefits for BGI. This clinical sample analyzing business is evaluated to be worth 10 billion RMB when BGI sold a part of its shares to raise capital for purchasing Complete Genomics in 2012 [2]. Testing services is at present mainly about reproductive health, such as prenatal noninvasive genetic testing (PNGT) for chromosome trisomy abnormalities and testing for other hereditary diseases. BGI also offers testing for infectious diseases, blood diseases and cancer [5].

The last part is testing for individual consumers, which is a downstream goal for BGI. The goal is to use personal genetic information in clinical routine practice when the cost of sequencing and analysis becomes low enough and the diagnostic usefulness of the information has been improved sufficiently.

Concerns have been raised in general about quality controls in various companies involved in testing of clinical samples in China. From September 2013 to February 2014, the National Health and Family Planning Commission (NHFPC, former Ministry Of Health) and the China Food and Drug Administration (CFDA) issued several public notifications to strengthen the governance of genetic testing in China. As a result, part of BGI’s commercial testing services was suspended for using an unapproved sequencer in clinical diagnosis [6, 7]. Later on, the CFDA reopened a registration channel for genetic testing service and equipment used in clinics. BGI submitted their materials, and is still waiting for approval [8]. Concerns have also been raised about the lack of separation of the various activities, in particular about samples/data management, and the risk of data appropriation. There is also a general worry that “unfettered access to the genetic building blocks of humanity” may actually come true [9].

There is no doubt that BGI carries out reputable research. However, overemphasis on the research part may lead to a lack of scrutiny of more questionable practices in clinical genetic testing. In this paper, we will mainly focus on the ethical issues arising from the research part, and the challenges for China to develop an appropriate regulatory framework for these types of research activities. This is not only an important domestic concern but also an issue of great international significance as BGI is a major player in many important international research projects. Table 1 summarizes some of the notable projects BGI is involved in.

**Table 1:** **Noted research of BGI under the blueprint**

Noted research	Classification under the blueprint
HGP, HapMap, 1000 Genomes, ICGC Project	Scientific research
BGI & Merck 2011, BGI & Danish 2011, BGI & START 2013	Scientific research, sequencing & analyzing service, further collaborations
The China National Genebank (Shenzhen)	Scientific research and further collaborations
PNGT in hospitals,	Medical/clinical services
The 1000 Rare Disease Project with CHOP	Scientific research, medical/clinical services with potential individual services in the future
IQ research	Scientific research with unclear applications
Cloud storage to manage data crossing boundaries	Unclear

Here are some examples of BGI’s international research project collaborations. As part of BGI’s Million Human Genomes Project, BGI and the Children’s Hospital of Philadelphia (CHOP) launched the 1000 Rare Disease Project in 2011 [10]. Scientists from CHOP stated that “BGI’s capabilities and expertise in whole genome sequencing and analysis, combined with Children’s Hospital’s extensive biobank and expertise in clinical phenotyping, will allow scientists and clinicians to harness the power of large, detailed data sets to improve the lives of patients and families” [11]. They also claimed that the collaborations would “have the capacity to basically read out and sequence every child that comes in to CHOP in the near future” [12]. In the same year, BGI and Merck announced to establish a collaboration to focus on the discovery and development of biomarkers and genomic technologies [13]. BGI and Danish organizations also initiated cooperation for cancer vaccines and the Danish genome research project, which may develop and patent commercial vaccines and establish a database of genetic variations in the Danish population [14]. In 2013, BGI collaborated with South Texas Accelerated Research Therapeutic (START) to launch the San Antonio 1000 Cancer Genome Project, which aims to combine heritable variations with clinical phenotypes to facilitate personalized medicine development. This project is privately funded. START is responsible for patient recruitments, sample and clinical data collections from local hospitals and BGI is responsible for the genome sequencing [15]. Furthermore, in 2011, after approval by the China National Development and Reform Commission and other national commissions, BGI-Shenzhen built and operated the China National Genebank (Shenzhen) [16], to “establish a Biological Resource Bank, an Information Database and a Network domestically and across the globe for providing a powerful support for genomics-related scientific research and applications to promote the goals of biodiversity protection and sustainable development” [17].

Over the past few years BGI has sequenced the genomes of many important species from rice to the giant panda, human disease samples, some rare diseases [10], and the first Asian genome [18]. Up to 2012, BGI has sequenced 540 plant and animal genomes, 25,239 variant copies and 38,123 human samples [19]. BGI’s machines generate six terabytes of data each day [20] and a huge amount of data need to be handled across the country and overseas on its network. Cloud storage is being used to electronically share data [21]. An internal test conducted by BGI reveals that it merely takes 30 seconds to move a 24-gigabyte file between China and the USA [22].

Some of these projects have indeed raised concerns outside of China. In February 2013, The Wall Street Journal posted a news article titled: “A Genetic Code for Genius” [23] that reports a sensitive IQ study by BGI. 2,200 DNA samples, mostly from America, allegedly of the brightest people with IQs of 160 or higher were used for sequencing, in an attempt to find the genetic basis of IQ. A behavioral geneticist who engages in this research claims such understanding could help prepare support in advance for kids who are identified as having learning problems. The news article pointed out worries on how genetic data on IQ could be misconstrued or misused. “Research into the science of intelligence has been used in the past ‘to target particular racial groups or individuals and delegitimize them,….the reductionist and deterministic trends that still are very much present in the world of genetics would come to the forefront in a project like this” [23]. A catchy headline on BBC, “China’s designs to engineer genius babies” no doubt has added fuel to fire in this discussion [24].

These high-profile disputes in the mass media undoubtedly have raised concerns in the general public about the company’s operations and how it is regulated. In this paper, both for the sake of BGI’s own interests and for the good of the general public, we will focus on the question whether the Chinese regulatory infrastructure and its ethical institutions are appropriate enough to form the codes of conduct and guidelines for the sound practice of such a genetic giant.

### Regulatory issues

Before moving forward to the governance and bioethics s problems derived from BGI in the Chinese context, the basic Chinese biomedical research regulation framework will be briefly reviewed.

At the domestic level, in 2010 the National People’s Congress (NPC) passed the *Tort Liability Law of the People’s Republic of China* [25]. Besides domestic laws, the State Council, NHFPC, the Ministry of Science and Technology (MST) and CFDA have promulgated and revised a series of laws and regulations to construct a governance framework for biomedical research. The main laws and regulations that involve human genetic research and sample/data management are summarized in Table 2. With the rapid development of scientific research, some regulations have been updated or revised in recent years.

**Table 2:** **Laws and regulations on genetic research in China**

	Laws & Regulations
The Interim Procedures for Human Genetic Resources Management by NHFPC & MST in 1998	Covers research, development and transfer of genetic materials, rules and procedures of application and approval;
	Requires informed consent from donors and relatives for international collaborations but does not mention IRB review and specific content about informed consent;
	States that genetic resources and related information should be protected as state secrets of science and technology, does not mention sample reuse and research information feedback to individual donors.
The Good Clinical Practice by CFDA in 2003	To ensure that clinic trials of new medicines are scientifically reliable, protect subjects’ right and safety;
	conforms to *Declaration of Helsinki*, follows principles of justice, respect and beneficence/non-maleficence;
	Clear requirement to protect the benefits of subjects by appropriate IRB reviews and informed consent about the clinic trial;
	The definition of clinical trial refers research “on human body” that may be literally inapplicable to genetic research;
	The requirement of informed consent does not mention particular issues of consent for genetic research, which at least should include consent for future use of samples, feedback and withdrawal from research.
The Ethical Review of Biomedical Research Involving Human Subjects by NHFPC in 2007	Rules for duties and jurisdictions of IRB, clearly states the constitution of IRB;
	Stipulates ethical principles of IRB review with standard procedures;
	Clearly requires protection of privacy, subjects can quit at any time without any condition, re-consent shall be obtained if research protocol has been changed;
	The definition of research still literally problematic in genetic research context;
	Not provides details about feedback and withdrawal method that fits genetic research.
The Tort Liability Law of the People’s Republic of China by NPC in 2010	Protects legitimate rights and interests of civil subjects in general, explicitly ensures citizens’ right to life, to health as well as the right of privacy. Especially in the chapter of compensation for medical damage it notes health care organizations and medical professionals should protect patients’ privacy and shall bear the liability for tort if patients get damaged due to privacy leaking or the disclosure of patients’ medical records.

China has established a regulatory framework of research that by and large follows international guidelines. However, considering the actual complex practical contexts and new situations emerging with the rapid progress in genetic research, tensions always exist between regulations and implementations of these regulations. This is especially the case for issues related to sample/data collection and management where there are many unresolved controversies and every country has their own non-uniform technical and ethical criteria [26]. These tensions are magnified in a developing country like China that eagerly wants to boost its biomedical industry, but needs to balance that goal with how to conduct research with high integrity. As shown below, if regulations are too broad or abstract, there would be no binding power and the regulations would not have any practical impacts. On the other hand, if regulations are too rigid, scientific progress could be hampered. Occasionally, an ambiguous terminology in the regulations creates self-conflicting principles and renders the regulations difficult to interpret or unenforceable.

## Discussion

We have identified two issues of concern with regard to the Chinese genetic research regulations. First, since there are variations in the regulations between countries, there may be ethical inconsistencies in international collaborative projects when BGI has to follow the regulations from both the country of origin of the samples, and Chinese regulations. Second, although existing Chinese regulations apparently follow general international guidelines and principles, they are too broad to apply to substantive issues that may arise in current research practices. This may allow for some questionable practices. In addition, issues such as strategies of combining informed consent with rules for re-consent, withdrawal and feedback from research as well as ways the issue of genetic privacy raise special challenges in genetic research in China. This will be illustrated in the following discussion.

### An ambiguous term in the regulations

Before turning to more substantive issues, let us first discuss a terminological issue that exists in the current legislation. This terminological issue actually shows that policy makers might have simply adopted international regulations on general research practices without paying sufficient attention to the special challenges of genetic research on samples and data.

With regard to human sample collection, the *Interim Procedures for Human Genetic Resources Management* issued by NHFPC & MST in 1998, which covers genetic research, requires informed consent for international collaborations but does not mention the need for an IRB review [27]. Afterwards, the NHFPC promulgated the *Ethical review of biomedical research involving human subjects* in 2007, which requires IRB review for all biomedical research involving human subjects and their informed consent. However, in the definition part, it states its coverage extends to:

“…biomedical research involving human subjects and relevant technical applications that …using …ways to perform research on the human body for knowing… or …conducting experimental application activities on the human body by using medical care technology or product…”

This formulation is ambiguous. It seems that genetic research projects are exempt from this regulation and therefore exempt from IRB review if they only use detached samples that have been delinked from their original subjects according to the original legislation in 1998. However, even when using detached human samples/data, genetic research samples are clearly parts of the human body and therefore should still be classified as human subject research and be subjected to IRB review according to the legislation in 2007. In 2012 the initial draft of *the Regulation of the Human Genetic Resources* was published by the State Council for public comments. Although the regulation has not officially come into force yet, it would replace the former interim measures of 1998. The regulation officially requires IRB review for genetic research. In July 2013, NHFPC issued the initial draft edition of the *Administrative Measures of Medical Science and Technology Research Involving Human Subjects* for comments. The definition problem is still there, but this draft clearly classifies human genetic research as a “high risk” type of research which is subject to more strict reviews. Since the existing regulations leave the definition issue open, the question that arises from this situation is what review levels should be used to monitor genetic research before the new regulation takes effect. Moreover, if we take the regulation literally, the definition of research is also too narrow to cover psychological and behavioral studies, which means that this kind of research will fall outside the scope of the regulatory framework. These potential inconsistencies between various regulations need to be avoided, and serious thought ought to be given to how the Chinese regulations should distinguish human subject research from other types of research, and what oversight mechanisms are appropriate for different types of research on human subjects.

### Withdrawal and feedback from research

There seems to be a general trend towards increased requirements for informed consent in international regulations, requiring researchers to pay more explicit attention the protection of subjects. The *Interim Procedures for Human Genetic Resources Management* of 1998 states that informed consent is required for genetic international collaborations but provides for very general requirements, and blanket consent for future research. The *Ethical review of biomedical research involving human subjects* in 2007 clearly requires informed consent for all research and re-consent is needed if the protocol changes. It also states that subjects can quit at any time from the research. The regulation puts subjects in the center of research design and subjects are protected by means of IRB review and informed consent. Moreover, with the rise of genetic research and the importance of biobanks, samples/data instead of the human body are used more frequently in studies. In light of this, the initial draft of *the Regulation of the Human Genetic Resources* in 2012 places further specific emphasis on genetic research. The draft requires that the collection and storage of human genetic samples should abide by principles of autonomy and informed consent. Before sample collection, written informed consent shall be offered to the donor to explain the purpose, usage, potential health risks, interest-sharing plans, privacy protection and other necessary relevant information about the research. Subjects have the right to quit unconditionally at any time. It also requires that re-consent should be requested if samples are used for other purposes beyond the initial consent. Moreover re-consent is needed if genetic samples were originally collected for routine clinical diagnosis and treatment, or from blood banks, crime investigations, doping controls, funeral and forensic agencies not earmarked for genetic research, or used for other research that goes beyond the primary purpose, or for international cooperation.

Some may favor this draft in terms of its strict requirements for specific items of informed consent, but critics may think it still only dogmatically interprets the general biomedical international guidelines, and cannot successfully handle deeper and controversial dilemmas in genetic research practices. Samples/data usage is different from traditional human subject research or research on clinical patients in the sense that protocols or interventions are implemented not directly on the human body but rather on samples or data that have been detached from human subjects or patients. Such research is of low risk of direct harm to the original subjects/patients, but may harm them indirectly or run against their reasonable will if the relevant sensitive information is leaked or misused by the researchers. Furthermore, delinked samples/data may be used multiple times for other purposes which may expose participants to unknown risks. There is also a question on how to handle incidental findings in genetic research that may be important for the welfare of donors/subjects. The desire to know or not to know research results or feedback may vary between individuals according to their autonomy and preferences. Regulations that can accommodate reasonable variations in the views of diverse research subjects are therefore needed.

One empirical study of the opinions of some Chinese patients and the public towards problems about consent for the use of clinical leftover samples in research shows that among 64.7% respondents who are willing to donate for research, only 16.7% donors wish to keep the withdrawal option afterwards. Only 12% of the respondents accepted future research without specific consent and 74.3% respondents wanted to receive feedback of relevant research results [28]. These results are quite different from those reported in other countries [29], where generally a high number of research subjects accept future research without consent. It is interesting to point out that the percentage of those who wish to keep the withdrawal option and those who consent to future research without the need for re-consent are equally low. Therefore, there seems to be an inconsistency in these answers, i.e., we cannot simply conclude that Chinese subjects place a high or a low value on autonomy. The low number of people who accept future research without consent implies a high desire for control of what happens to their samples. On the other hand, the low number of people who wants to keep the withdrawal option implies a low desire for control of what happens to their samples. Whatever the empirical study concludes, it does at least illustrate the complexity of opinions about these issues, but existing national regulations do not say anything about issues related to feedback and withdrawal from research. This shows that the current regulations did not pay appropriate attention to the complex nature of samples/data usage in genetic research.

These issues have been a subject of intense discussion in the international bioethics community. There is evidence of a lack of international consensus on how one should handle samples/data in different systems and people have proposed different strategies with regard to informed consent [30]. Some experts propose that a one-time general consent with an opt-out model would be a preferential option for biobanks in China. The donor’s wish to withdraw from research can be guaranteed by the “opt-out” setting. How the samples are used in the biobank can be revealed to the donors in ways that are understandable for them [31]. Internationally, some have evaluated the ethical conditions that justify broad consent for future research and pointed out that a right to withdraw consent would be necessary [32]. Some considers the right to withdraw from research should be assessed on the basis of the potential harm to the subjects and points out that delinking should not be the only automatic permissible response to requests for withdrawal [33]. Others have analyzed how researchers should confront incidental findings and what obligations they owe to the research subjects in different circumstances [34–36].

On examining the regulations of biobanks outside of China, one can observe quite a flexible approach. For example, in the withdrawal procedures of the UK Biobank, there are options at different levels [37]: no further contact, no further access and no further use. They also allow sample/data to be disposed of according to the donor’s wish after considering questions about feedback of research. The donors are also offered options on the right to know/not to know about research results, and to what extent the researcher can use his/her samples/data. If such a flexible and layered withdrawal method could be adopted by an overseas counterpart, why should China adhere to the rigid unconditional withdrawal option?

A similar problem appears against feedback strategies of results from the research. The ethical review standard specification of the China National Genebank (Shenzhen) [38] states that unless samples and data have been delinked from the donor, the donor can withdraw from the study at any time unconditionally. Although it compares the “opt-in” and “opt-out” model of informed consent, it rules that IRB should check whether samples are used for purposes beyond what is stated in the original informed consent form, or without the donor’s consent and whether irretrievable delinkage is performed when samples/data circulate freely. The policy effectively guarantees all the subjects the right to quit anytime. However, once the samples/data have been delinked, they can be reused freely. This approach potentially suffers from two major drawbacks. Firstly, it seems that this clause bypasses and conceals the crucial problem: who has the right to make the delinking decision that may limit the usefulness of samples/data, the researcher, the IRB or the donors themselves? Secondly, have these delinked subjects been deprived of their right to extract their samples/data from the research database because an IRB thinks delinking is sufficient to protect subjects from potential harm? The question becomes more complex when donors in some countries could be given several withdrawal options but donors in China only are provided with a single one. This nonconformity may appear in IRB review when samples from multiple countries are analyzed in China. Could this nonconformity be deemed as ethically unacceptable by treating donors differently? Delinking data also gives rise to new problems. Delinked data may lose their scientific value because the patient’s medical history may reveal valuable information on the underlying genetic conditions. One might argue that donors should be made aware of this trade-off before signing on to the research where the only option of reusing the data is to delink it. In addition, what if significant results are found in a research project when investigators feel they have a strong obligation to trace the subjects back, but is then impossible because samples/data have been irreversibly delinked? The current regulation simply deals with whether samples/data are delinked or identifiable, but has neglected many other relevant ethical concerns. It ultimately does not really offer subjects and the public a chance to actively participate and receive feedback from research projects they may regard as important. Therefore, all the above information and options should be taken into consideration when designing a consent form for subjects/donors. The form should not just focus on informed consent, but should provide comprehensive information on withdrawal, delinkage, re-consent for new research purposes, as well as the feedback method of possible research findings.

### Genetic discrimination and risk of data leakage

Concerns have been raised about the sensitive nature of the research and the enormous amount of data that BGI handles, especially when there is a risk of genetic discrimination and breach of privacy. As shown above, BGI handles very sensitive research like the IQ study. BGI also uses Cloud storage for the massive amount of data they generate. This may allow easy transfer of data between researchers, but increases the risk of data breaches. Appropriate measures to guarantee data security need to be implemented. Even after a particular research project has been completed, the information from the project may still be valuable to further research so there is a need to store all data securely. Data leakage of such highly sensitive personal genetic information could lead to serious privacy violations.

Some may argue the issues of genetic discrimination arising from genetic research in China is an unnecessary worry. However, lawsuits concerning genetic discrimination in the mainland and Hong Kong in recent years seem to indicate otherwise. In 2001, a judicial decision about genetic discrimination in employment in Hong Kong caught some public attention [39]. Three young men who have no signs of mental problems were rejected to be employed as local civil servants because of a history of mental illness in their parents. They sued relevant departments based on *the Disability Discrimination Ordinance*. After considering the conditions of the plaintiffs and the inherent requirement of the positions, the court ruled that “it was unlawful for the civil service to discriminate in employment, for the sake of public safety, against people with a family history of mental illness” [39]. A similar lawsuit happened in city of Foshan near Guangzhou in mainland China in 2009. Three men were refused the positions in the local civil service because they are carriers of the thalassemia gene. They sued the local Human Resources and Social Security Bureau but failed. However, genetic testing has been removed from the list of the local civil servant enrollment health examination in May 2011 [40]. These two cases raise the issues of who could justifiably use personal or their relatives’ medical/genetic information and in what circumstances, and how individual privacy can be protected in this era of rapid genetics progress and biotechnology advances.

As early as 2000, the UK Genetics and Insurance Committee approved that insurance coverage could be affected by the result of Huntington's predictive test [41]. In 2011, a moratorium on the use of predictive testing for insurance policies with high payouts was extended until 2017 [42]. In 2008 the US *Genetic Information Nondiscrimination Act* was passed to protect against damage for the insured due to his/her genetic test results [43]. In China, in 2010 after the Foshan genetic discrimination lawsuit, proposal for initiating the development of a genetic discrimination law has been submitted during the National People's Congress and the Chinese Political Consultative Conference [44]. So far no laws have been formally legislated. However, the exposure draft of *the Regulation of the Human Genetic Resources* in 2012 requires that organizations or individuals not to conduct human genetic research and development that may result in discrimination. Anyone who breaks this law shall be punished and each IRB is empowered to review and monitor genetic research within its institution. This regulation shows the right intention to reduce genetic discrimination by strengthening ethical review. However, this brings on new questions regarding the functions of an IRB. Traditionally, the duty of an IRB is to review samples/data management for the subjects who participate in a specific research project. Its function would not extend to protect those who suffer discrimination or stigmatization due to the long-term impacts. To fulfill this mission, the IRB has to identify what kinds of discrimination a particular genetic research may cause and has to assess the possibility of risks. It is also not clear at what risk level should the IRB prohibit a specific research project. In some situations, a mere possibility would prohibit all genetic research. How can various IRBs reach consistent conclusions when a multi-national genetic research project is carried out in different cultural contexts? What criteria should be used to guide the assessment? These are open questions that can hardly be solved by merely providing a single sentence on the requirement in legislation. Some concrete feasible methods need to be discussed. Otherwise, the good intention behind the regulation will not have any meaningful impact in practice.

Genetic information leaks may have more far reaching impacts than information leaks in clinics or other social situations. Many countries have introduced legislations to address this concern. Given the global nature of genetic research now, one should consider whether it is morally necessary to achieve the goal of global protection against genetic discrimination and protection of privacy by setting the same sets of criteria along the whole samples/data pipeline, or whether we should leave each country the discretion to make their own rules. It also poses the practical issue of how to harmonize different regulations among countries involved in research and how to ensure those who use genetic sample/information adhere to a uniform requirement. Given the global reach of companies like BGI, regulatory protection of genetic information in China is not just of interest within the country, but also to researchers worldwide.

Recent developments highlight the need for increased attention to privacy issues. In 2013, researchers at Cambridge, Massachusetts reported that the identity of an individual who contribute their DNA sequence for research project can be traced back by using publicly available internet resources [45]. This means that there is “A potentially serious loophole that could allow anyone to unmask the identities of people who contribute their DNA sequences to some research projects” [45]. Recognizing the reality of this threat, some people think it is imperative to disclose to research subjects that “it is unlikely that their identities can be kept hidden if their genetic data are revealed” [46]. More extreme caution is warranted because a major genomic data leak may just be a matter of time, “the question is not how to prevent a leak but how to mitigate the fall-out” [46]. It would definitely bring unknown detrimental risks to the subjects’ privacy. This can also damage genetic research in the long run, because loss of trust from subjects and the potential legal liabilities for compensations may deter investment in further research.

## Summary

From the above, we can see that there are some apparent gaps in the Chinese regulatory system regarding bioethical issues related to genetic research. These issues may partially be due to the nature of genetic research and partially stem from the strategy of simply adopting a general international guideline into a new practice without careful consideration of the needs of genetic research. However there isn’t a perfect ready-made ethical solution to be adopted by any country, as many of these issues are still controversial in China and other countries. Controversies exist on questions like withdrawal from research, information feedback, and how to protect privacy. Ethical approaches may vary greatly in different projects carried out by different countries and institutions. An influential player in the biomedical field that processes massive amounts of global samples/data like BGI and China as a whole has an obligation to consider these problems. Countries that send their samples to China for analysis also have an obligation to follow the developments on these ethical issues in China. Finally, we should all take part in a larger conversation about the development of more concrete international standards to ensure scientifically sound and ethically harmonious genetic research in the future.

## Abbreviations

BGI: The Beijing Genomics Institute
CFDA: The China Food and Drug Administration
HGP: The Human Genome Project
HapMap: The International Human HapMap Project
1000 Genomes: The International 1000 Genomes Project
ICGC Project: The International Cancer Genome Project
PNGT: Prenatal noninvasive genetic testing
IRB: Institutional Review Board
CHOP: The Children’s Hospital of Philadelphia
START: The South Texas Accelerated Research Therapeutic
NPC: The National People's Congress
NHFPC: The National Health and Family Planning Commission
MST: The Ministry of Science and Technology
RMB: Ren Min Bi.
